# Supplementary material for: MetaQTL: a package of new computational methods for the meta-analysis of QTL mapping experiments
Source: BMC Bioinformatics. 2007 Feb 8;8:49. doi: 10.1186/1471-2105-8-49 (PMC1808479; doi:10.1186/1471-2105-8-49)
Supplement: Additional File 7 — MetaQTL Package : jar file and tutorial. This Zip archive contains both the MetaQTL JAR file and the files of the tutorial. [file 1471-2105-8-49-S7.zip › org.inra.metaqtl/doc/org/thalia/bio/entity/package-summary.html]

org.thalia.bio.entity


|  |  |  |
| --- | --- | --- |
| **Overview** \| **Package** \| Class \| **Use** \| **Tree** \| **Deprecated** \| **Index** \| **Help** | |  |
| **PREV PACKAGE**   **NEXT PACKAGE** | **FRAMES**    **NO FRAMES**     **All Classes** |  |


---

## Package org.thalia.bio.entity

| **Interface Summary** | |
| --- | --- |
| **IBioConstants** | Class Description Here |
| **IGeneticMapProperties** | Class Description Here |

| **Class Summary** | |
| --- | --- |
| **AlleleContainer** | Class Description Here |
| **BioEntity** | Class Description Here |
| **BioEntityCollection** | Class Description Here |
| **BioEntityContainer** | Class Description Here |
| **CrossPopulation** | Class Description Here |
| **GeneticMap** | Class Description Here |
| **GroupContainer** | Class Description Here |
| **Individual** | Class Description Here |
| **IndividualContainer** | Class Description Here |
| **LGroup** | Class Description Here |
| **Locus** |  |
| **LocusContainer** | Class Description Here |
| **Marker** | Class Description Here |
| **Ontology** |  |
| **OntologyTerm** |  |
| **Population** | Class Description Here |
| **QTL** | Class Description Here |

---


|  |  |  |
| --- | --- | --- |
| **Overview** \| **Package** \| Class \| **Use** \| **Tree** \| **Deprecated** \| **Index** \| **Help** | |  |
| **PREV PACKAGE**   **NEXT PACKAGE** | **FRAMES**    **NO FRAMES**     **All Classes** |  |


---
